# Nanoelectronics: Materials, Devices and Applications

**DOI:** 10.3390/nano14211716

**Published:** 2024-10-27

**Authors:** Chunchang Wang

**Affiliations:** Laboratory of Dielectric Functional Materials, School of Materials Science & Engineering, Anhui University, Hefei 230601, China; ccwang@ahu.edu.cn

## 1. Introduction

The semiconductor industry is facing concerns regarding the saturation of Moore’s Law [1]. To address this challenge, great efforts have been made to develop nano-scaled functional electronic devices [2,3,4,5] in order to form a rapidly developing field of nanoelectronics that focuses on the manipulation and control of nano-scale materials and devices. Due to the quantum size effect, electrons in nanomaterials and devices exhibit many novel properties, several of which have attracted great attention from researchers in various fields. Nanoelectronics bridge the gap between traditional electronics and quantum mechanics, achieving unprecedented miniaturization and enhanced functionality. The exploration of materials, devices, and their applications in nanoelectronics has achieved significant breakthroughs in various industries, from healthcare to energy systems. It is widely believed that nanoelectronics will replace microelectronics as the mainstay of information technology in the coming decades, which will have a profound impact on human life.

## 2. An Overview of Published Articles

This Special Issue comprises ten research articles, two communication articles, and three review articles covering a variety of fields:

Polymers in nanoelectronics. Nordendorf and their coauthors reported that dispersing ferroelectric LiNbO_3_: Fe nanoparticles in liquid crystal–polymer composites could lower transition temperatures and birefringence, thus enhancing the electro-optic performance [6]. For the optimization of the process of integration with Cu films, Ustad et. al. investigated the adhesion of photosensitive polyimide (PSPI) films with different substrates, including Si, SiN, SiO_2_, Cu, and Al. The PSPI films is stable on Cu substrate at high temperatures of up to 350 °C. This will be helpful for new packaging applications, such as a 3D IC with a Cu interconnect [7].

Devices in nanoelectronics. Zhao et al. simplified the fabrication process of thin-film transistors (TFTs) prepared by high-power impulse magnetron sputtering (HiPIMS) at room temperature via a two-step deposition pressure process. Compared with traditional uniform channels, this process has the advantages of balancing the high mobility and low threshold voltage of TFTs [8]. Xie et al. presented a ZnO TFT memory utilizing self-assembled Au nanocrystals. The memory exhibits excellent memory performance, including a program/erase window of 9.8 V, a 29% charge loss extrapolated to 10 years, and remarkable endurance characteristics. Their work indicates that the fabricated TFT memory has great potential for practical applications [9]. Alam et al. used a simple, non-toxic, environmentally friendly, and water-driven method to manufacture gate dielectrics on silicon substrates and successfully integrated the In_2_O_3_/HfO_2_ TFTs. The device exhibits the best electrical performance at an optimized annealing temperature. Their results demonstrate the potential application of aqueous solution technology for future low-cost, energy-efficient, large-scale, and high-performance electronics [10]. Park et al. analyzed the effects of interface traps on the output characteristics of an inversion mode *n*-channel GaN Schottky barrier (SB)-MOSFET using TCAD simulations. The simulation results demonstrated that the shallow trap affected the device’s switching performance and photo-response characteristics significantly, while the deep trap had a significant effect on the device’s on-state performance [11]. Dou et al. designed and fabricated high-frequency bulk acoustic wave (BAW) resonators based on Al_1-x_Sc_x_N-based piezoelectric films with Sc concentrations as high as 30%. The fabricated BAW resonators demonstrate a large effective electromechanical coupling of 17.8% at 4.75 GHz parallel resonant frequency and excellent temperature stabilit with the temperature coefficient of frequency of −22.9 ppm/°C [12]. Wu et al. investigated the effect of atomic layer deposition (ALD)-derived Al_2_O_3_ passivation layers and annealing temperatures on the interfacial chemistry and transport properties of the sputtering-deposited Er_2_O_3_ high-k gate dielectrics on Si substrate. Their work shows that the ALD-derived Al_2_O_3_ passivation layer remarkably prevents the formation of the low-k hydroxides generated by moisture absorption of the gate oxide and greatly optimizes the gate dielectric properties. They achieved the lowest leakage current density of 4.57 × 10^−9^ A/cm^2^ and the smallest interfacial density of states of 2.38 × 10^12^ cm^−2^ eV^−1^ in the Al_2_O_3_/Er_2_O_3_/Si MOS capacitor [13].

Energy-harvesting in nanoelectronics. Zhao et al. fabricated a self-powered triboelectric nanogenerator (TENG) based on fish scales. The fish-scale TENG is a kind of flexible, wearable, and self-powered triboelectric nanogenerator showing great prospects in regard to healthcare and body-information monitoring [14]. Zheng at al. fabricated a ternary dielectric rotating triboelectric nanogenerator (TDR-TENG) based on TiO_2_/WO_3_ dual-band electrochromic material. The TDR-TENG can convert mechanical energy from the environment into electrical energy to obtain a high output of 840 V, 23.9 μA, and 327 nC [15]. Chakraborty et al. fabricated a novel bio-based TENG comprising PDMS/α-Fe_2_O_3_ nanocomposite film and a processed human-hair-based film. The TENG harvests the vibrating energy and solar energy simultaneously by the integration of triboelectric technology and photoelectric conversion techniques. Their work provides a new approach towards self-powered photo-detection while developing a propitious green energy resource for the circular bio-economy [16]. Wang et al. discussed the implementation of smart materials in TENGs: classification, design, function collaboration, and applications. They finally highlighted the challenges and outlooks in this field [17]. 

Electrons in nanoelectronics. Tian et al. demonstrated that the two-dimensional electron gas (2DEG) on the (100) KTaO_3_ (KTO) surface undergoes a semiconductor–metal transition under the illumination of visible light. Their results deepen the understanding of the photoinhibition effect of 2DEG semiconductor on the KTO surface and contribute to the exploration of the photoinduced modulation effect of 2DEG on the KTO surface [18]. Bangolla et al. reported the photoconduction properties of tungsten disulfide (WS_2_) nanoflakes obtained by the mechanical exfoliation method. The WS_2_ photodetector exhibits superior performance with responsivity in the range of 36–73 AW^−1^ and a normalized gain in the range of 3.5–7.3 × 10^−6^ cm^2^ V^−1^ at a lower bias voltage of 1 V. The results suggest that WS_2_ nanostructures are of potential as a building block for novel optoelectronic device applications [19]. Shi et al. found that the La_0.5_Na_0.5_TiO_3_ addition in (0.65BiFeO_3_–0.35BaTiO_3_) composites can improve the electrostrain properties due to the phase boundary effect. They obtained a good thermal stability of electrostrain with fluctuation η = 31% in a wide temperature range of 25–180 °C in the sample with a La_0.5_Na_0.5_TiO_3_ doping level of 4% mole. This work provides an implication for designing high-temperature piezoelectrics and stable electrostrain materials [20]. In the review article by Slimani et al., the authors highlighted the latest advancements in photonic curing for perovskite materials, hole transport layers, and electron transport layer materials. They emphasized that the significance of these advancements for perovskite solar cells could further highlight the importance of this research and underline its essential role in creating more efficient and sustainable solar technology [21].

Sensors and transducers in nanoelectronics. Yao et. al. reported that the (In + Nb) co-doped HfO_2_ ceramics, Hf_1−x_(In_0.5_Nb_0.5_)_x_O_2_ with x = 0.005, exhibit a superior humidity sensing performance. The good performance of the HfO_2_-based humidity sensor was ascribed to the defects created by doping, which improves the adsorption capacity for water molecules [22]. Polachan et al. presented a review of the physics of the body–electrode interface in on-body sensing and communication applications. They commented on how the body–electrode interface distorts signals and how these distortions affect biopotential sensing and human body communication [23]. 

## 3. Conclusions

Our Special Issue, although limited in theme, may promote and accelerate research on materials, devices, and applications in nanoelectronics. As research in this field progresses, we can expect even more groundbreaking developments in the near future.
